# Identification of internalin-A-like virulent proteins in *Leishmania donovani*

**DOI:** 10.1186/s13071-016-1842-5

**Published:** 2016-10-21

**Authors:** Ishita Mukherjee, Abhijit Chakraborty, Saikat Chakrabarti

**Affiliations:** Structural Biology and Bioinformatics Division, Council for Scientific and Industrial Research (CSIR) - Indian Institute of Chemical Biology (IICB), Kolkata, West Bengal India

**Keywords:** Cell invasion, LRR proteins, Inl-A-like proteins, Homology-based search, HMM-profile based search, *Leishmania donovani*

## Abstract

**Background:**

An active immune surveillance and a range of barriers to infection allow the host to effectively eliminate microbial pathogens. However, pathogens may use diverse strategies to subdue such host defences. For instance, one such mechanism is the use of leucine-rich repeat (LRR) proteins by pathogens (microbial) to cause infection. In this study, we aimed at identifying novel virulence factor(s) in *Leishmania donovani*, based on the possibility of lateral gene transfers of bacterial virulence factor(s) to *L. donovani*.

**Methods:**

Rigorous homology searching protocols including Hidden Markov Model (HMM) and BLASTp based searches were employed to detect remote but significant similarities between *L. donovani* proteins and bacterial virulence factors.

**Results:**

We found that some *L. donovani* proteins are similar to internalin-A (Inl-A) protein of *Listeria monocytogenes*, a surface LRR protein that helps mediate host cell invasion by interacting with E-cadherin on the cell membrane. However, to date, no such invasion mechanism has been reported in *Leishmania donovani*, the causative agent of visceral leishmaniasis. Moreover, a comparative LRR motif analysis of *L. donovani* Inl-A-like proteins against the Inl-A protein of *L. monocytogenes* revealed existence of characteristic consensus LRR regions, suggesting a reliable evolutionary relationship between them. Further, through rigorous three dimensional (3D) modeling of *L. donovani* Inl-A-like proteins and subsequent molecular docking studies we suggest the probability of human E-cadherin binding with the *L. donovani* Inl-A-like proteins.

**Conclusions:**

We have identified three potential candidates (UniProt ID: E9B7L9, E9BMT7 and E9BUL5) of Inl-A-like LRR containing proteins in *L. donovani* with the help of systematic whole genome sequence analysis. Thus, herein we propose the existence of a novel class of Inl-A-like virulence factor proteins in *L. donovani* and other *Leishmania* species based on sequence similarity, phylogenetic analysis and molecular modelling studies in *L. donovani*.

**Electronic supplementary material:**

The online version of this article (doi:10.1186/s13071-016-1842-5) contains supplementary material, which is available to authorized users.

## Background

*Leishmania* spp. (family Trypanosomatidae) are intracellular protozoan parasites that when transmitted to a mammalian host can cause a range of infectious diseases, collectively referred to as leishmaniasis. These parasites have two morphologically distinct variants during their life-cycle, a promastigote form in phlebotomine sand flies and an amastigote form in mammalian cells [1]. A complex array of processes is involved in the attachment and invasion of host cells, initially mediated by the promastigotes and subsequently by the amastigotes. In general, many proteins of *Leishmania* spp. have been identified as possible virulence factors. For instance, lipophosphoglycan and leishmanolysin are important in attachment, invasion and intracellular survival of the parasites [2, 3]. In addition, the leucine-rich repeat (LRR)-containing proteins proteophosphoglycan and parasite surface antigen 2 also participate in parasite attachment and invasion of host cells [2]. Moreover, the A2 protein is important for the survival of the pathogen in visceral organs [4]. Amastin, amastin-like surface protein and cysteine proteases are other groups of proteins that attribute virulence to this group of parasites [5, 6]. Thus, *Leishmania* spp. can utilize several polypeptide and polysaccharide ligands while interacting with the host cells and some of these interactions may be synergistically or alternatively involved in receptor-mediated endocytosis. A number of receptors on host cells, which include neutrophils, macrophages, monocytes or dendritic cells may interact with these parasites. However, once phagocytosed, *Leishmania* spp. can generally establish long term infections within phagosomal vesicles by subverting host immune responses [1]. Depending on the host’s immune response and the species that infects it, such an infection may result in the clinical presentation of cutaneous (*L. major*, *L. mexicana*, *L. amazonensis* and *L. braziliensis*), muco-cutaneous (*L. braziliensis* and *L. panamensis*) or visceral leishmaniasis (*L. donovani* and *L. infantum*) [1]. In particular, nearly 20,000–50,000 deaths occur annually from visceral leishmaniasis and about 200,000–400,000 new cases of visceral leishmaniasis occur worldwide each year. Nearly 90 % of these new cases occur in six countries including India [7].

In an approach to develop better intervention strategies against visceral leishmaniasis, the *L. donovani* genome has been sequenced, annotated and compared with other *Leishmania* spp. to understand the genome, its natural variation and genetic population structure [8]. Previously, genome comparisons of *L. major*, *L. infantum* and *L. braziliensis* showed great conservation of synteny and identified only a small number of genes to be differentially distributed [9] and these species-specific genes may be responsible for the difference in pathogenesis between the species. Further, comparison of the *L. donovani* genome with the other *Leishmania* species showed substantial genetic differentiation at the species level between the genomes of *L. infantum* and *L. donovani* [8]. Similarly, comparison between *L. donovani* clinical isolates (from Nepal and India) provided insights into emerging modes of drug resistance among them [8]. Therefore, a range of virulence factors leading to internalization and/or persistence of *L. donovani* in phagolysosomes and other visceral organs, have been elucidated. However, increasing instances of visceral leishmaniasis with emerging drug resistance is still persisting, especially in some parts of the Indian subcontinent. Hence, identification of new and more effective drug targets in *L. donovani* remains to be an important issue.

In this study, we aimed at identifying novel virulence factor(s) and their possible invasion mechanism(s) in *L. donovani.* In this respect, we assumed that lateral gene transfers of bacterial virulence factor(s) to parasitic eukaryotes (*L. donovani*) is likely, since a recent report suggested the possibility of such transfers particularly of enzymatic genes belonging to core pathways such as the amino acid and sugar metabolism [10, 11]. Thus, preliminarily we searched for *L. donovani* proteins bearing significant resemblance to bacterial virulence factors or their virulence domains in particular with the help of the virulence factors database [12]. We identified a few *L. donovani* proteins possibly having an Internalin-A (Inl-A) like domain similar to *Listeria monocytogenes* Inl-A, which is a surface LRR protein that helps mediate host cell invasion by interacting with E-cadherin on the cell membrane [13]. We devised a strategy referred to as forward and reverse search analysis comprising of profile *versus* sequence (Hidden Markov Model (HMM) [14] based) and sequence *versus* sequence (BLASTp [15]) comparisons to confirm the existence of such Inl-A-like remote orthologs in *L. donovani*. With the help of this search strategy we identified remote but significant similarities between certain *L. donovani* proteins (*L. donovani* Inl-A-like proteins) and LRR containing Inl-A protein from *Listeria monocytogenes*. We next asked questions like, whether these remote orthologs shared a significant evolutionary relationship with Inl-A, or what is the extent of similarity between such *L. donovani* Inl-A-like proteins and their bacterial counterparts? Moreover, it would also be interesting to study whether these *L. donovani* Inl-A-like proteins share similar subversion mechanism in host cells as *L. monocytogenes* Inl-A? We addressed the first question by studying the orthologs of these proteins and by comparing the signature leucine rich repeat (LRR) motifs between the two sets of proteins. Whereas the second question was assessed by homology modeling of *L. donovani* Inl-A-like proteins and their subsequent docking studies with the protein interaction partner (human E-cadherin [hEC1]) of their bacterial ortholog (Inl-A). Based on these analyses we suggest the existence of a new group of virulence factors capable of employing a yet to be known mode of host invasion mechanism in *L. donovani*.

## Methods

### Dataset collection

For the purpose of this study, we have collected *L. donovani* proteome (8083 protein sequences) from TriTrypDB (release 8) [16, 17]. Selected protein sequences of *L. donovani* were also obtained from UniProt database [18] and three dimensional coordinates of the template protein were obtained from the protein data bank (PDB) [19].

### Search for virulence factors in *L. donovani*

In order to search for virulence factors in *L. donovani*, the virulence factors database (VFDB, containing 34,230 complete sequences) was downloaded [12, 20]. Each of the *L. donovani* sequences was compared against this database using BLASTp algorithm (Basic Local Alignment Search Tool, BLAST, for searching protein databases using a protein query) [15]. Proteins satisfying the threshold, E-value of ≤ 1e^-8^, sequence identity of ≥ 20 %, query protein (*L. donovani*) length coverage of ≥ 25 % and subject protein (VFDB) length coverage of ≥ 40 % were selected as possible bacterial virulence factor-like proteins.

### Bacterial virulence factor-like sequences in *L. donovani*

**Fig. 1:**
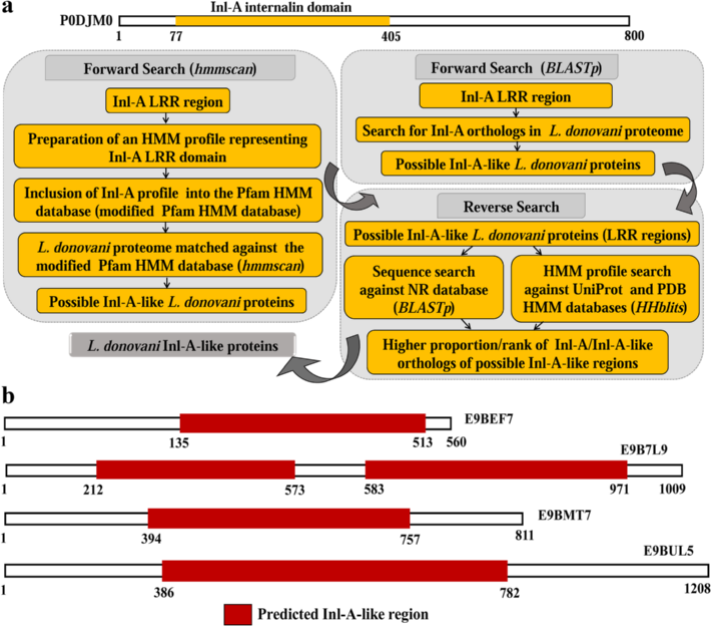
Basic methodology for identification of *L. donovani* Inl-A-like proteins. **a** Outline of the basic search strategy to identify *L. donovani* Inl-A-like proteins. **b** Identified *L. donovani* Inl-A-like proteins with the help of ‘forward search analysis’ followed by ‘reverse search analysis’. *Abbreviations*: Inl-A, Internalin-A; LRR, Leucine Rich Repeat; HMM, Hidden Markov Model; BLAST [15], Basic Local Alignment Search Tool; HHblits, HMM-HMM-based lightning-fast iterative sequence search tool [30]

Existence of *L. donovani* proteins with reasonable similarity with bacterial Inl-A-like virulence proteins indicated the probability that these *L. donovani* proteins might be distant orthologs of Inl-A. To ascertain this similarity, we employed additional approaches referred to as forward and reverse search analysis (Fig. 1).

#### Forward search analysis

Initially, a BLASTp search was performed against the *L. donovani* proteome using the *L. monocytogenes* Inl-A LRR region (UniProt ID: P0DJM0 [region: 77–405]) as query [15]. Protein segments bearing an E-value of ≤ 1e^-10^, query length coverage of ≥ 60 % and sequence identity of ≥ 20 % with Inl-A LRR region were presumed to be similar to the Inl-A domain.

A hidden Markov model (HMM) profile representative of Inl-A was prepared to perform a sequence *versus* profile search. For this purpose, close homologs of Inl-A were determined *via* BLASTp search against all bacterial genome databases (homolog selection criteria: E-value of ≤ 1e^-6^, query length coverage of ≥ 70 %, sequence identity of ≥ 40 %) [15, 21]. The Inl-A-like region (± 10 residues) was extracted from these bacterial orthologs and representative non-redundant (< 95 % identical) sequences were selected for further analysis [22, 23]. Inl-A and its bacterial orthologs (81 sequences) were aligned with an iterative refinement method (MAFFT-L-INS-I version 7) giving preference to local alignment such that the conserved LRR motif regions are well aligned [24, 25]. The resultant multiple sequence alignment (MSA) was utilized to prepare HMM profile *via* HMMER 3.0 [14, 26]. This Inl-A profile along with other known Pfam [27, 28] family profiles (modified Pfam-A database) was used as profile database against which each *L. donovani* protein was scanned for similarity. For this purpose, we utilized the *‘hmmscan’* program of HMMER 3.0 package that allows one to search for similarity using sequence(s) against a profile (HMM) database [14, 26]. It was assumed that sequence(s)/domain(s) having an E-value of ≤ 1e^-8^, target coverage of ≥ 50 %, sequence identity of ≥ 20 %, conservation of ≥ 70 % with the Inl-A profile are probably Inl-A-like sequences. Among these proteins, the ones having considerably high scoring predicted Inl-A LRR domains (domain score ≥ 110) were selected for reverse search analysis. Moreover, an HHpred [29] analysis using HHblits (HMM-HMM-based lightning-fast iterative sequence search) algorithm [30] and maximum accuracy (MAC) alignment (MAC realignment threshold = 0.3) was done for each of these proteins against the protein data bank (PDB) HMM database to define the LRR regions in these proteins more reliably.

#### Reverse search analysis

Proteins from *L. donovani* which showed similarity to bacterial Inl-A sequence *via* forward search approaches were further matched against the NCBI non-redundant (NR) sequence database [31, 32] using BLASTp [15, 33] and against the UniProt and PDB databases using HHblits [30, 34]. For sequence-sequence comparisons using BLASTp, sequences having an E-value of ≤ 1e^-6^, query length coverage of ≥ 70 %, sequence identity of ≥ 20 % with the query were considered as homologous. Similarly, for the (HMM) profile-(HMM) profile comparison using HHblits search, sequences having probability of ≥ 97 % were assumed to be homologous. Here, probability reflects the percentage score that a database match is a true positive based on a comparison of non-homologous (negative) and homologous (positive) domain pairs in an all-against-all comparison of structural classification of proteins (SCOP) [35]. These proteins, which share similarity with the predicted Inl-A-like regions in the *L. donovani* proteins, were then further filtered to represent only non-Kinetoplastida and well-characterized proteins. This was done such that we can get a better estimate of a homologous relationship between these *L. donovani* proteins and other bacterial proteins or internalins. Such homologous proteins were then ranked based on the E-value and only the top 25 subject hits were taken for further analysis to determine if these *L. donovani* proteins bear any significant similarity to LRR proteins or Inl-A. Thus, *L. donovani* proteins showing significant sequence similarity with the bacterial Inl-A protein(s) in both forward and reverse search analysis were considered as *L. donovani* Inl-A-like proteins.

### Evaluating sequence based similarity between bacterial Inl-A and *L. donovani* Inl-A-like proteins

Bacterial Inl-A and *L. donovani* Inl-A-like proteins were aligned pairwise using the Needleman-Wunsch algorithm available in EMBOSS (v6.2.0) [36] for the computation of sequence identities between them. Sequences sharing an E-value of ≤ 1e^-6^, query length coverage of ≥ 70 % and sequence identity of ≥ 45 % with *L. donovani* Inl-A-like proteins and bacterial Inl-A protein from NCBI non-redundant (NR) database [31, 32] were taken as close orthologs of *L. donovani* Inl-A-like proteins and bacterial Inl-A proteins, respectively. MSA containing the close orthologs of *L. donovani* Inl-A-like and bacterial Inl-A protein having sequence identity of ≤ 95 % was used for creating a phylogenetic tree by the Randomized Axelerated Maximum Likelihood (RAxML version 8.1.6) [37] program utilizing the maximum likelihood algorithm. The generated tree was edited in ETE (version 2.3.8) [38].

Further, the similarity between Inl-A LRR regions and the predicted LRR regions in *L. donovani* Inl-A-like proteins were compared using the HHalign [39] program. The HMM profile for each LRR region of *L. donovani* Inl-A-like proteins was constructed as mentioned before (criteria for selecting orthologs: E-value ≤ 1e^-6^, query length coverage ≥ 70 % and sequence identity ≥ 30 %). Additionally, distantly orthologous bacterial internalin sequences were assimilated (bacterial internalin sequences having sequence identities in the range of 20–30 % with *L. monocytogenes* Inl-A were determined using PSI-BLAST version 2.2.28+ [40]). Similar HMM-HMM comparison between LRR regions from distant internalin sequences and Inl-A LRRs was done.

### Homology modeling

Three dimensional (3D) coordinates for each of the selected *L. donovani* Inl-A-like proteins were predicted by MODELLER 9.15 [41–44] with the help of *L. monocytogenes* Inl-A [PDB ID: 1O6S, chain A] as template and structure-sequence alignment provided by the HHpred server [29]. 3D models were ranked on the basis of energy parameters like DOPE score and structures were validated using VERIFY3D [45, 46], RAMPAGE [47]. Certain selected models were further refined and loop modeling was performed in MODELLER 9.15 to predict most plausible 3D models with least stereo-chemical violations. The homology models were visualized and analyzed in chimera 1.10.1 [48]. Since calcium (Ca^2+^) is important for the interaction of Inl-A with hEC1 [13], Ca^2+^was coordinated with the corresponding residues in the *L. donovani* models. In models where corresponding Ca^2+^coordinating [49] residues were missing, other similar residues within 5 Å were considered and ligand Ca^2+^ was incorporated in the models with a coordination sphere of 2.45 Å (similar to Inl-A structure) in MODELLER 9.15. These models were further filtered based on DOPE score and validated using previously mentioned structure validation tools.

### Docking and selection of predicted interaction pose

The modeled *L. donovani* Inl-A-like structures were superimposed onto the template Inl-A structure and residues within 5 Å of hEC1 were taken as possible interacting residues while for hEC1 residues known to interact with Inl-A were taken as possible interacting residues. Docking of *L. donovani* Inl-A-like proteins with hEC1 was guided with this a priori interaction information while performing molecular docking *via* multiple programs namely, PatchDock [50, 51], HADDOCK 2.0 (High Ambiguity Driven protein-protein Docking) [52–54] and ClusPro 2.0 [55–57], respectively. Similarly, re-docking of Inl-A with hEC1 was also performed with these multiple programs to study whether the docking programs are capable of capturing the native Inl-A-hEC1 interaction conformation reliably (Additional file 1: Figure S1). PatchDock [50, 51] allows geometric shape complementarity matching with the help of geometric hashing and pose-clustering techniques and FireDock [58] allow further refinement of the complexes. Top 100 solutions from PatchDock-based on the receptor-ligand geometric score were clustered according to root mean square deviation (RMSD) in chimera (version 1.10.1) [48] to determine largest docked clusters. Solutions present in each cluster were further refined in FireDock [58]. HADDOCK [52–54] is mainly a data driven (ambiguous interaction restraints) rigid-body docking process with solvent based refinement of complexes where the predicted interacting residues were taken as active residues and passive residues were defined automatically around the active residues. ClusPro [55–57] utilizes Fast-Fourier Transform (FFT)-based approach and correlation between binding site free energy attractor and cluster size to provide an approximation of the native binding conformation. While docking in ClusPro the residues, which possibly interact, were assumed to share attractive forces.

Assuming that clusters having higher number of similar frames with more average negative docking scores (better energetics) are more likely to possess the best possible interaction pose between two proteins, top three clusters (Cluster I, Cluster II and Cluster III) were selected from each docking program for further evaluation. A representative frame from each cluster was aligned with Inl-A crystal structure and ligand RMSD (l-RMSD) was calculated in chimera v1.10.1 [48]. The docking scores (FireDock refined glob score, HADDOCK score, ClusPro balanced score) of these ranked clusters from each program were plotted with l-RMSD for better enumeration of similar interaction poses. Here, FireDock refined glob score represents the binding energy score of the complexes calculated considering desolvation energy (atomic contact energy, ACE), van der Waals interactions, partial electrostatics, hydrogen and disulfide bonds, π-stacking and aliphatic interactions, rotamer’s probabilities etc. [50, 51, 58], whereas, HADDOCK score is a weighted sum of intermolecular electrostatic, van der Waals, desolvation, ambiguous interaction restraints (AIR) energies, and a buried surface area (BSA) term [52–54]. Moreover, ClusPro balanced score is given as the sum of terms representing shape complementarity (attractive and repulsive interactions), electrostatic and desolvation contributions [in terms of pairwise potentials like DARS (Decoys As the Reference State) and ACP (atomic contact potential)] [55–57].

Since a consensus pose predicted from all three programs utilizing different scoring functions ascertains more confidence to such an interaction pose, these solutions were considered as the best docked conformations. Finally, the free energy of binding of *L. donovani* Inl-A-like proteins and hEC1 as well as the probable hydrogen-bonding interaction patterns of these complexes (the selected best docked conformations) were determined using PISA (available in ccp4mg (version 2.8.1) [59]). Moreover, residues involved in probable hydrophobic interactions were determined with the help of Protein Inter-Chain Interaction (PICI) web server [60].

## Results

### Similarity between bacterial virulence factors and *L. donovani* proteins

**Fig. 2:**
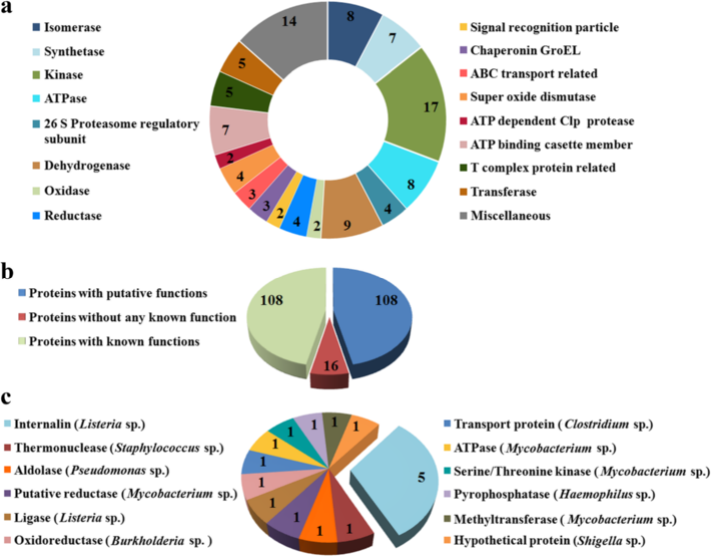
Search for bacterial virulence factor-like proteins in *L. donovani.* The virulence factor database (VFDB) [12] was searched to identify whether any *L. donovani* proteins bear significant similarity (E-value ≤ 1e^-8^, sequence identity ≥ 20 %, query length coverage ≥ 25 %, subject length coverage ≥ 40 %) to bacterial virulence factor proteins. **a** Functional distribution of *L. donovani* proteins listed in the UniProt database [18] bearing significant similarity to bacterial virulence factors. **b** Distribution of *L. donovani* proteins having significant matches with the VFDB [12] proteins according to the search. **c** Predicted functional distribution of *L. donovani* proteins without any known functions as determined by similarity to VFDB [12] proteins

An in-depth analysis aiming to establish possible existence of bacterial virulence factor-like proteins was undertaken based on the observation that lateral gene transfer from microbes to Trypanosomatidae may have played a role in their evolution [10, 11]. Since, we were interested in identifying remote orthologs, carrying representative virulence factor-like domains in the *L. donovani* proteome, the threshold for selection of similar proteins was implemented accordingly. On searching the virulence factor database (VFDB) [12], we found 232 *L. donovani* proteins that possess significant sequence similarity with bacterial virulence factor-like proteins (Fig. 2b). However, functions for some of these proteins (108) are already known (Fig. 2a), whereas some have putative functions and still others have no known functions. Based on the similarity to VFDB proteins predicted functions were assigned to *L. donovani* proteins without functional classification (Fig. 2c; Additional file 2: Table S1). It was interesting to note that 32 % of the proteins with unknown functions were found to be similar to bacterial (*L. monocytogenes*) Inl-A proteins possibly sharing an internalin domain.

### Establishment of distant orthology between *L. donovani* proteins and bacterial Inl-A

Identification of homologs can be done based on database searches using sequence-sequence comparisons. In order to ascertain the orthologous relationship between bacterial internalin-A and *L. donovani* proteins, we utilized a strategy referred to as forward and reverse search analysis as outlined in Fig. 1. In the forward search analysis, BLASTp [15] search against *L. donovani* proteome suggested two parasite proteins (UniProt ID: E9B7L9, E9BMT7) to be Inl-A-like (Table 1). However, a number of studies have suggested that profile-profile and sequence-profile-based searches are more sensitive than sequence-sequence searches in identifying remote orthology since profiles include more information than one sequence alone about a related family of proteins [15, 39, 61]. For instance, given a multiple sequence alignment (MSA) of bacterial Inl-A homologs (only for LRR regions) probability theory was used to define an HMM profile containing position specific information about the degree of conservation of the internalin domain. Correspondingly, while comparing the *L. donovani* proteome with the modified Pfam-A database (containing the Inl-A HMM profile) using ‘*hmmscan*’ [14, 26–28] a range of domains were predicted including LRRs in many *L. donovani* proteins. Since we were interested in establishing the existence of Inl-A-like proteins in *L. donovani,* we selected proteins having an Inl-A-like LRR region for further analysis. Thus, we identified five *L. donovani* proteins (UniProt ID: E9BUL5, E9BEF7, E9B7L9, E9B8L0, E9BMT7) sharing significant similarity with the Inl-A profile (Table 2). This finding is intriguing because the Inl-A LRR region is mainly involved in interaction with human host cell receptor E-cadherin (hEC1) and in turn plays a major role in invasion of the host cells in *Listeria* pathogenesis [62].

**Table 1:** Forward search analysis (BLASTp)

Subject^a^ ID (Gene symbol)	Alternate protein IDs (Subject)^a^ (Ensembl, RefSeq GI, UniProt)	E-value^b^	Query^c^ (aligned region)	Query^﻿﻿c﻿﻿^ coverage (%)	Subject^a^ (aligned region)	Subject^a^ coverage (%)	Sequence identity (%)
LdBPK_030010.1	emb|CBZ31242; gi|398009536; E9B7L9	3.94e^-19^	10–342	96.8	439–831	38.95	25.25
LdBPK_311630.1	emb|CBZ36565.1; gi|398020181; E9BMT7	6.35e^-14^	16–339	94.19	301–684	47.35	24.68

Predicted distant Inl-A orthologs in *L. donovani* proteome identified based on BLASTp [15] search

^a^Subject: corresponds to *L. donovani* proteins which are probably Inl-A-like

^b^E-value (expect-value): the average expected number of non-homologous proteins with a score higher than the one obtained for the database match; E-values closer to 0 are statistically significant

^c^Query: corresponds to Inl-A LRR region

**Table 2:** Forward search analysis (*hmmscan*)

Query^a^ ID (Gene symbol)	Alternate protein IDs [Query]^a^ (UniProt, Ensembl, RefSeq GI)	Subject^b^ coverage (%)	Query^a^ length	Domain score^c^	c-Evalue^d^	i-Evalue^e^	Query^a^ (Aligned region)	Sequence identity (%)	Conservation^f^ (%)
LdBPK_365070.1	E9BUL5, emb|CBZ38944|, gi|398024920	81.49	1208	117	3.30e^-37^	6.10e^-34^	394–776	28.18	75.26
LdBPK_191670.1	E9BEF7, emb|CBZ33633, gi|398014298	55.74	560	141.6	1.10e^-44^	2.00e^-41^	265–526	26.98	77.78
LdBPK_030010.1	E9B7L9, emb|CBZ31242|, gi|398009536	56.6	1009	117.4	3.10e^-37^	4.50e^-34^	202–467	23.05	75.78
LdBPK_030010.1	E9B7L9 emb|CBZ31242|, gi|398009536	60.64	1009	145	1.30e^-45^	1.90e^-42^	563–847	28.52	78.15
LdBPK_051200.1	E9B8L0, emb|CBZ31583, gi|398010218	59.57	396	146	5.70e^-46^	9.40e^-43^	77–356	29.06	76.23
LdBPK_311630.1	E9BMT7, emb|CBZ36565, gi|398020181	72.13	811	213.8	1.60e^-66^	2.70e^-63^	394–732	30.65	84.21

Predicted distant Inl-A orthologs in *L. donovani* proteome identified based on *hmmscan* [14, 26] against a modified Pfam database [27, 28] (including an Inl-A profile)

^a^Query: corresponds to *L. donovani* proteins

^b^Subject: corresponds to Internalin-A internalin domain profile

^c^Domain Score: the calculated domain score for the best aligned profile (Inl-A)-sequence (*L. donovani*) matched states of the Inl-A-like internalin domain predicted in *L. donovani* proteins

^d^c-Evalue (conditional E-value): measures the statistical significance of each domain, given that the target sequence is a true homolog. It is the expected number of additional domains one would find with a domain score this high in the set of sequences reported in the top hits list, if those sequences consisted only of random non-homologous sequence outside the region that sufficed to define them as homologs

^e^i-Evalue (independent E-value): the significance of the sequence in the whole database search, if this were the only domain one had identified

^f^Conservation: estimation of identical or similar matching states over the alignment length

**Fig. 3:**
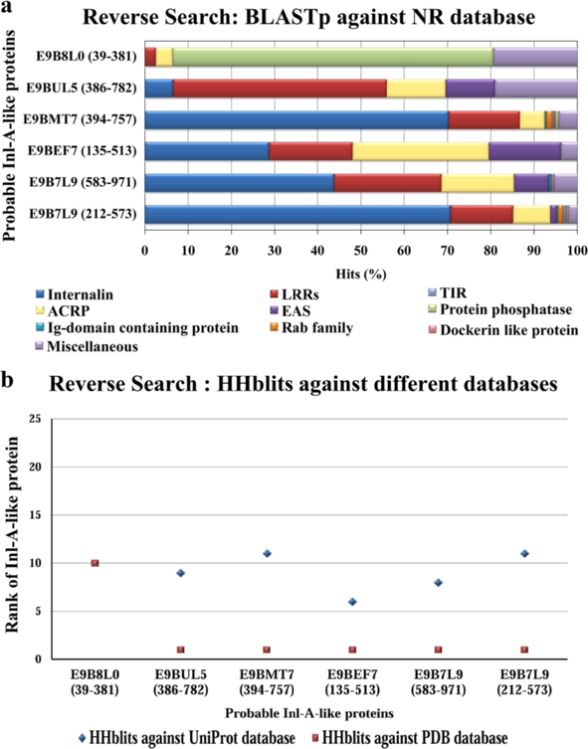
Reverse search analysis. **a** The percentage of major functional classes of homologous proteins (hits) obtained from BLASTp [15] search for each probable Inl-A-like region of *L. donovani* proteins. **b** The ranks of Inl-A/Inl-A-like proteins which share similarity with each of the probable Inl-A-like region of *L. donovani* proteins based on HHblits (HMM-HMM-based lightning-fast iterative sequence search) search against PDB and UniProt HMM databases [30, 34]. Ranks of the Inl-A and Inl-A-like proteins within top 25 similar proteins are shown

Four of these *L. donovani* proteins contain one Inl-A LRR-like region while E9B7L9 possesses two possible Inl-A LRR-like regions. Further, HMM-HMM-based search was performed using HHblits algorithm [30] and MAC realignment was done for each *L. donovani* Inl-A-like protein to delineate the LRR regions in these proteins more appropriately (Additional file 3: Table S2). The reverse search analysis, compared the predicted Inl-A-like LRR regions of the identified *L. donovani* proteins against different sequence databases [NCBI non redundant database (NR), UniProt database] [18, 31, 32] and structure database (PDB) [19] to confirm whether these predicted Inl-A-like LRR regions bear reliable similarity to LRR-containing proteins or internalins. These comparisons were done with the help of BLASTp [15] against NR [31, 32] database and HHblits against UniProt and PDB HMM databases [30, 34]. We found indications that E9BMT7 (region: 394–757) as well as E9B7L9 (region: 212–573) are highly similar to different internalins as ~71 and ~70 %, respectively of their significant orthologous ‘hits’ retrieved *via* BLASTp [15] belong to internalins (Fig. 3a). Similarly, E9B7L9 (region: 583–971), E9BUL5 (region: 386–782), E9BEF7 (region: 135–513) also contain LRR rich regions, which bear similarity to internalins. Moreover, the HMM-HMM-based search results when ranked according to E-value show that Inl-A and Inl-A-like proteins are among the top ranked candidates for *L. donovani* Inl-A-like regions (Fig. 3b). However, during the reverse search analysis, it was found that E9B8L0 (region: 39–381) is less likely to possess an internalin domain. Thus, based on the search for virulent proteins in the VFDB database [12] and the forward as well as reverse search analyses utilizing different sequence-sequence, sequence-profile and profile-profile comparison methods we propose that E9BUL5, E9BEF7, E9B7L9 and E9BMT7 proteins possibly contain Inl-A-like LRR regions within their sequence.

It is also possible that similar Inl-A-like proteins are present in other trypanosomatids and/or Kinetoplastida. Using similar approaches we have identified 8 non-*Leishmania* probable internalin-A-like proteins within trypanosomatids and/or Kinetoplastida proteome (see Additional file 4: Figure S2 for details). Similarly, identification of 13 leishmanial orthologs of *L. donovani* Inl-A-like proteins indicates that these Inl-A-like proteins are not unique to *L. donovani* and it is likely that an Inl-A-like class of proteins is present in *Leishmania* spp. and other kinetoplastids (Additional file 4: Figure S2).

### Establishment of LRR motif similarity between bacterial Inl-A and *L. donovani* Inl-A-like proteins

**Fig. 4:**
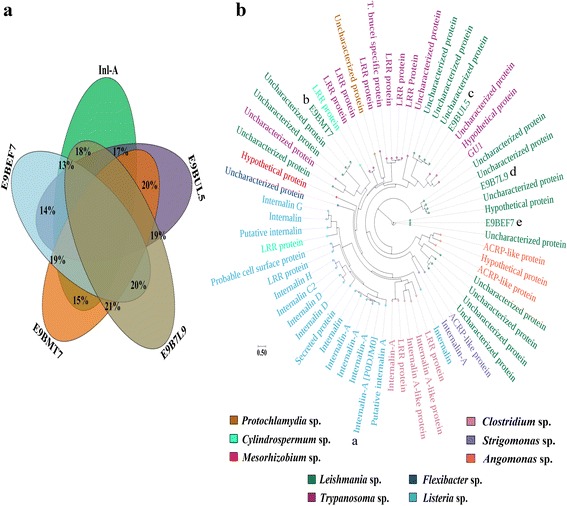
Comparison of internalin-A (Inl-A) and *L. donovani* Inl-A-like proteins. **a** Pairwise sequence identities between *L. monocytogenes* Inl-A and *L. donovani* Inl-A-like complete sequences. **b** Orthologs of Inl-A and *L. donovani* Inl-A-like proteins were compared to establish the extent of orthology they share. The generated tree in circular layout shown here exemplifies the distant orthology between Inl-A (a) and E9BMT7 (b), E9BUL5 (c), E9B7L9 (d), E9BEF7 (e), respectively

**Fig. 5:**
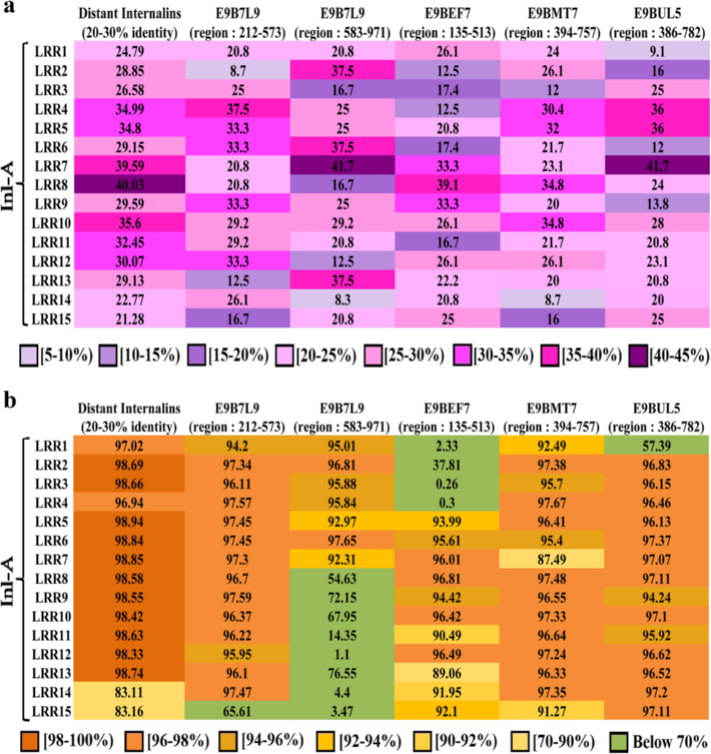
LRR motif similarity between internalin-A (Inl-A) and *L. donovani* Inl-A-like proteins. **a** The pairwise sequence identities between each Inl-A LRR and the corresponding predicted LRR of *L. donovani* Inl-A-like proteins along with the average LRR sequence identities between Inl-A LRRs and distant internalin-like sequences. **b** Probability of similarity [calculated based on (HMM)profile-(HMM)profile comparisons] between Inl-A LRRs with the corresponding predicted LRRs of *L. donovani* Inl-A-like proteins are shown along with average probability of similarity with respect to distant internalin-like sequences. Probability values >90 % indicate a homologous relationship that is either globally homologous or locally similar in structure to the Inl-A LRRs

Pairwise sequence identities and phylogenetic analysis suggest distant orthology between Inl-A orthologs and *L. donovani* Inl-A-like proteins (Fig. 4a, b). Hence, distant orthologs (sequence identity between 20 and 30 %) of *L. monocytogenes* Inl-A proteins were identified and average sequence identities of the equivalent LRR motifs (LRR1-15) among them were calculated for comparison*.* A varied range (8–42 %) of sequence identities was observed for most of the LRR motifs between *L. donovani* Inl-A-like proteins and *L. monocytogenes* Inl-A sequence (Fig. 5a). Further, HMM profiles for each LRR motif extracted from the bacterial Inl-A distant orthologs and *L.donovani* Inl-A-like proteins were constructed and compared across these two sets of sequences (Fig. 5b). This exercise indicated that the probability of a orthologous relationship between bacterial Inl-A LRRs and predicted LRRs of E9B7L9 (region: 212–573), E9BMT7 (region: 394–757) and E9BUL5 (region: 386–782) are reasonably high, since a profile-profile comparison yielded probability of ≥ 90 % and an E-value of ≤ 1e^-5^ for all the LRRs except one which had significant E-value but probability ≤ 90 %. However, profiles of predicted LRRs for E9B7L9 (region: 583–971) and E9BEF7 (region: 135–513) when compared with profiles of bacterial Inl-A LRR motifs either do not show significant E-values (E-value of ≥ 1e^-5^) or probabilities (probability ≤ 90 %) for some of the LRRs (Fig. 5b). Since, for a sequence to be like Inl-A, it must have a reasonable degree of similarity in all of the LRRs, the Inl-A-like proteins from *L. donovani* were further pruned at this stage and only E9B7L9 (region: 212–573), E9BMT7 (region: 394–757) and E9BUL5 (region: 386–782) were selected for further study.

### Homology modeling of *L. donovani* Inl-A-like proteins

Based on the previous analysis, we found that similarity between E9B7L9, E9BMT7 and E9BUL5 and *L. monocytogenes* Inl-A (P0DJM0) falls within the twilight zone (20–30 % sequence identity) of sequence similarity. However, the other analyses strongly indicate that E9B7L9 (region: 212–573), E9BMT7 (region: 394–757) and E9BUL5 (region: 386–782) probably have Inl-A-like LRR repeat stretches. Thus, we prepared three-dimensional (3D) model structures for these proteins based on the LRR region of *L. monocytogenes* Inl-A crystal structure (PDB ID: 1O6S, chain A). Since, HMM-HMM comparison yields high quality alignments in the twilight zone of sequence similarity, we used the previously generated alignment from HHpred for template-based modeling [29, 39].

**Fig. 6:**
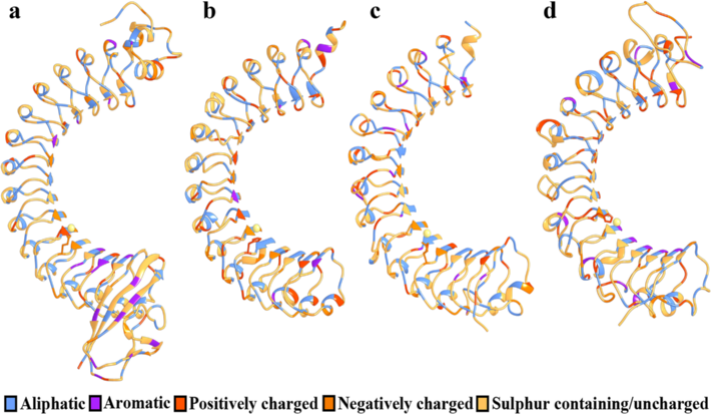
Homology models of *L. donovani* Inl-A-like proteins. Ribbon representation of *L. monocytogenes* Inl-A internalin domain (Uniprot ID: P0DJM0, PDB ID: 1O6S, chain A) (**a**) and of homology models of E9B7L9 (region: 212–573) (**b**), E9BMT7 (region: 394–757) (**c**) and E9BUL5 (region: 386–782) (**d**). Amino acid residues are coloured based on the nature of residues

Generally, internalins have an N-terminal cap domain, a LRR-domain (22 amino acid repeats), an inter-repeat region (IR), and C-terminal repeats. The internalin domain (region 36–496 of mature Inl-A) is comprised of an α-helical domain (residues: 36–78), LRR domain (residues: 79–414) and an immunoglobulin-like domain (residues: 415–495). In particular, the LRR domain has 15 full and a half 22 residue repeats with each repeat containing a β strand (xxLxL, L: leucine, valine, or isoleucine; x: any amino acid) followed by a loop (xxNxLxx), a 3_10_- helix (LxxLx), and a second loop (xLxxL). These repeats create a right-handed solenoid with a stretch of conserved aliphatic hydrophobic residues and an asparagine directed toward the solenoid core [13]. The *L. donovani* Inl-A-like proteins mostly have 23 residue repeats in contrast to the 22 residue repeats present in *L. monocytogenes* Inl-A. 3D homology models were prepared for E9B7L9 (region: 212–573), E9BMT7 (region: 394–757) and E9BUL5 (region: 386–782). Parameters describing these model structures are outlined in Table 3. In general, the predicted 3D structures have 15 LRR repeats each of which have a β sheet, followed by a loop, a helical region and another loop creating a solenoid. The solenoid created by these repeats exhibits a similar pattern of aliphatic hydrophobic residues towards its core (Fig. 6).

**Table 3:** Validation of homology models of *L. donovani* Inl-A-like proteins

*L. donovani* Inl-A-like proteins		E9B7L9	E9BMT7	E9BUL5
Predicted Inl-A-like Region		212–573	394–757	386–782
Sequence similarity index				
Whole Sequence Identity with Inl-A (UniProt ID: PODJMO)		18 %	15 %	16.8 %
LRR Region Sequence Identity with Inl-A (UniProt ID: PODJMO)		21 %	20 %	22 %
LRR Region Sequence Similarity with Inl-A (UniProt ID: PODJMO)		41.7 %	41.2 %	33.4 %
Structure Similarity Index				
Root Mean Square Deviation (RMSD) [Å] with Inl-A (PDB ID: 1O6S, chain A)		1.2	1	1.5
Stereo-chemical Evaluation				
Ramachandran Plot [47]	Favored	320 (88.9 %)	319 (88.1 %)	334 (84.6 %)
	Allowed	37 (10.3 %)	39 (10.8 %)	53 (13.4 %)
	Outlier	3 (0.8 %)	4 (1.1 %)	8 (2.0 %)
Verify3d [45, 46]		Passed (93.65 % of the residues had an averaged 3D–1D score > = 0.2)	Passed (91.21 % of the residues had an averaged 3D–1D score > = 0.2)	Passed (88.92 % of the residues had an averaged 3D–1D score > = 0.2)

### Docking of *L. donovani* Inl-A-like proteins with internalin receptor E-cadherin (hEC1)

Since, homologous proteins may share protein interaction partners [63] here we have explored the possibility that the *L. donovani* Inl-A-like proteins might possibly interact with hEC1 as the *L. monocytogenes* Inl-A is known to interact with hEC1. E-cadherin fills the cavity created by the curved repeat domain of Inl-A and most of the repeats bind hEC1 with the help of hydrogen bonds but LRR3 and LRR10 do not bind, while LRR12-15 have a patch of aromatic amino acids forming extensive hydrophobic interactions with hEC1 [13].

**Fig. 7:**
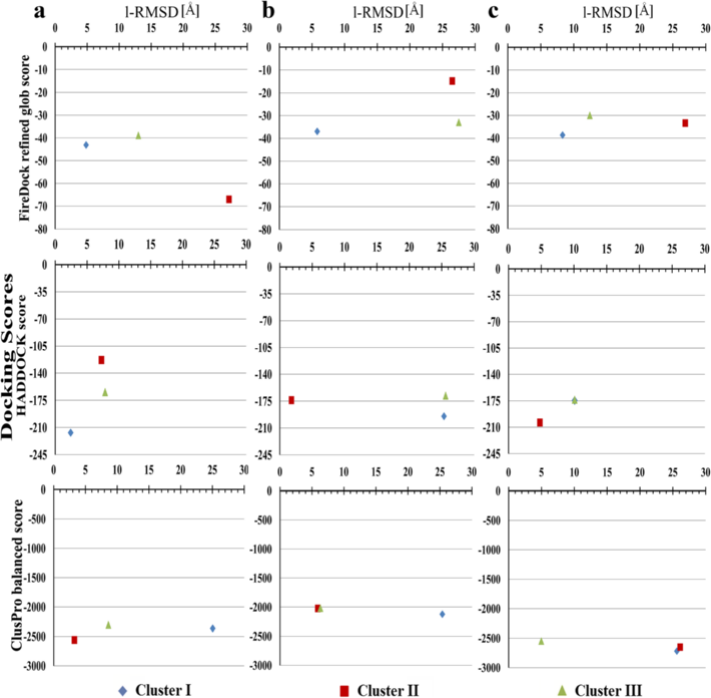
Molecular docking of *L. donovani* Inl-A-like proteins with human E-cadherin (hEC1). Directed docking solutions from each program were ranked and compared to identify consensus poses from all three programs (PatchDock [50, 51] followed by FireDock [58] refinement, HADDOCK [52–54] and ClusPro [55–57]). Average docking scores (FireDock refined glob scores, HADDOCK scores and ClusPro balanced scores) for the top three significant clusters (Cluster I, Cluster II and Cluster III) are plotted against the ligand root mean square deviations (RMSD) between the crystal complex (PDB ID: 1O6S) and representative docked poses of hEC1 with the *L. donovani* Inl-A-like proteins **a** E9B7L9 (region: 212–573), **b** E9BMT7 (region: 394–757) and **c** E9BUL5 (region: 386–782)

**Fig. 8:**
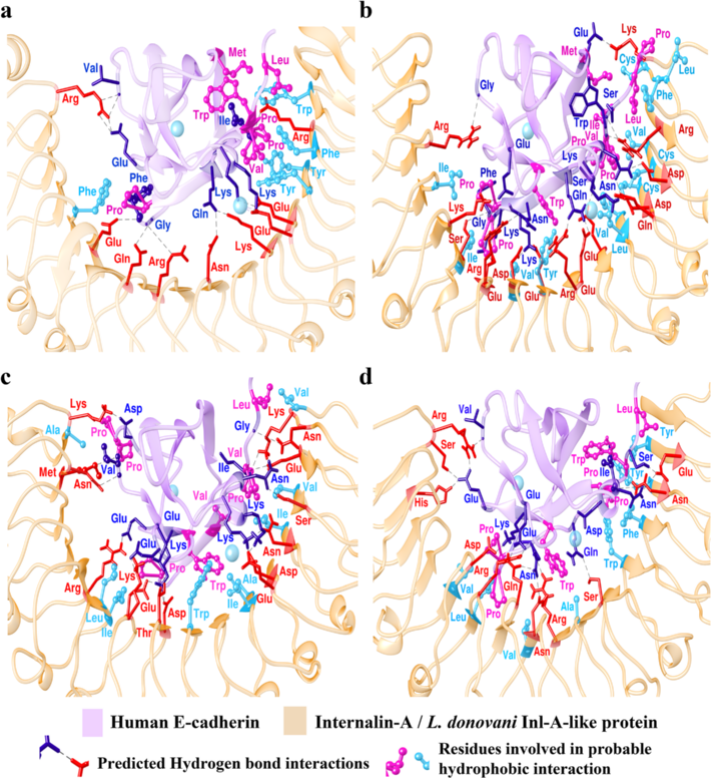
Probable docking conformations of hEC1 with *L. donovani* Inl-A-like proteins. **a** *L. monocytogenes* Inl-A internalin domain crystal structure in complex with hEC1 [PDB ID: 1O6S]. **b**-**d** Representative best-docked conformation of *L. donovani* Inl-A-like proteins with hEC1 similar to Inl-A-hEC1 interaction for E9B7L9 (region: 212–573) (**b**), E9BMT7 (region: 394–757) (**c**), and E9BUL5 (region: 386–782) (**d**). Key interacting residues probably involved in forming hydrogen bond interactions or hydrophobic interactions in E9B7L9-hEC1, E9BMT7-hEC1 and E9BUL5-hEC1 complexes are also shown. Ranked solutions from each program having ligand RMSD (l-RMSD) with Inl-A crystal structure within 10 Å were compared and best poses as predicted by HADDOCK [52–54] are shown here as representatives. In cases where hydrogen bond forming residue overlaps with hydrophobic interaction forming residue precedence is given to hydrogen bond color code while the residue is shown in ball and stick conformation to indicate that it is probably involved in hydrophobic interaction as well

We have used different approaches (varying protocols and scoring functions) for docking *L. donovani* Inl-A-like proteins with hEC1. Thus different scoring functions with various parameters allow us to evaluate the docked conformations, whereas ranking clusters based on higher number of similar frames is also suggestive of most likely interaction conformation between two proteins. Similar poses of *L. donovani* Inl-A-like proteins and hEC1 among the top three possible interaction poses (representative poses from clusters with higher number of frames or better docking scores namely Cluster I, Cluster II and Cluster III) were identified from the docking solutions of all the programs (Additional file 5: Table S3). Quality of protein-protein interaction predictions is generally assessed based on the Critical Assessment of Predicted Interactions (CAPRI) evaluation criteria, which utilize fraction of native contacts (Fnat) and ligand root mean square deviation (l-RMSD) to evaluate the performance of docking programs. In general, Fnat is determined by counting the number of contact residues that are retrieved in the predicted conformations between the two proteins with respect to the reference structure. Similarly, l-RMSD determined by calculating the RMSD on the backbone atoms of the smaller protein after fitting the model and the reference on the backbone atoms of the larger protein can be used to determine near native predictions. However, CAPRI criteria dictate 0.1 ≤ Fnat < 0.3 and l-RMSD ≤ 10 Å as acceptable predictions; 0.3 ≤ Fnat < 0.5 and l-RMSD ≤ 5 Å as medium quality predictions; and Fnat ≥ 0.5 and l-RMSD ≤ 1 Å as high quality predictions [52, 64]. Extending this paradigm based on the l-RMSD of the docked *L. donovani* Inl-A-like proteins with Inl-A structure we were able to postulate the likelihood of possible interaction between *L. donovani* Inl-A-like proteins and hEC1. Docked conformations when compared to *L. monocytogenes* Inl-A structure in complex with hEC1 were found to be within 5 Å for E9B7L9 (region: 212–573) and within 10 Å for E9BMT7 (region: 394–757) and E9BUL5 (region: 386–782), respectively (Fig. 7). These findings suggest the possibility of such interaction between *L. donovani* Inl-A-like proteins and hEC1. Further, the free energies of complex formation are -7.4, -8.8 and -10.4 kcal/mol for E9B7L9 (region: 212–573), E9BMT7 (region: 394–757), and E9BUL5 (region: 386–782) with hEC1, respectively, while it is -4.4 kcal/mol for Inl-A-hEC1 complex. However, on exploring the probable interaction forces that make these complexes stable we found hydrogen bond interactions as well as hydrophobic interactions in the predicted complexes of E9B7L9 (region: 212–573)-hEC1, E9BMT7 (region: 394–757)-hEC1 and E9BUL5 (region: 386–782)-hEC1 docked complexes (Fig. 8b–d; Additional file 6: Tables S4 and S5). Thus, all the molecular docking based observations indicate that the selected *L. donovani* Inl-A-like proteins might interact with hEC1 in a similar manner as *L. monocytogenes* Inl-A (Fig. 8).

## Discussion

*Leishmania donovani*, an intracellular protozoan, is one of the major pathogens causing visceral leishmaniasis worldwide [7]. In this context, previous studies have elucidated a number of proteins participating in the establishment of this infection, such as lipophosphoglycan, leishmanolysin, parasite surface antigen 2, A2 protein, amastin, amastin-like surface protein, cysteine protease B, etc. [2–6]. Genome sequencing studies and annotations may predict additional virulent proteins involved in this process [8]. During the establishment of parasitic infections, a number of different proteins may contribute to host-pathogen interactions. Therefore, herein we used sequence-based studies and phylogenetic analysis to identify potential virulence factors in *L. donovani* based on similarity to other known bacterial virulence factors. This study proposes the existence of an internalin-A-like class of proteins in *L. donovani*. Moreover, further studies in Kinetoplastida also indicated the presence of Inl-A-like class of proteins in other *Leishmania* spp. Elucidation of such a class of Inl-A-like virulence factors in *L. donovani* and/or other *Leishmania* spp. may provide a better understanding of the mechanism of infection(s) mediated by *Leishmania* spp. or *L. donovani* in particular*.*

*Leishmania* spp. as avirulent or metacyclic promastigotes can interact with different receptors like complement receptors (CR1 and CR3), mannose receptor (MR), fibronectin receptors (FnRs) on primary macrophages and dendritic cell-specific intercellular adhesion molecule-3-grabbing non-integrin (DC-SIGN) on dendritic cells for invasion [65]. However, they interact with Fc gamma receptors (FcγRs) in primary macrophages and dendritic cells for phagocytosis in amastigote stage [65]. It is plausible that these Inl-A-like proteins in *Leishmania* interact with E-cadherin or other receptors structurally similar to E-cadherin (like cadherin superfamily members) on host cell surface to promote their internalization. However, one might question the plausibility of such an interaction depending on whether cadherin is widely expressed on the skin or whether any of these leishmanial Inl-A-like proteins are expressed during initial stages of infection to be able to subsequently interact with host cell surface receptors. In particular, CDH1 (E-cadherin) is widely expressed in the skin, specifically in epidermal cells, langerhans cells, keratinocytes and melanocytes [66, 67]. Further, in this regard based on literature studies we have found that a close *L. major* homolog of E9BMT7 (95 % identical to E9BMT7) is present in the exosomes secreted in the midgut of the sand fly that are co-egested in the inocula with the parasite during the insect’s bite [68]. This observation indicates that leishmanial Inl-A-like proteins may be expressed in the promastigote stage and can possibly interact with host cell receptors to promote cell invasion during the initial stages of infection. However, direct experimental evidence to support this hypothesis, for instance, lower infectivity on knock down of these protein(s) or binding affinity assessment, on actual receptor or cadherin binding is lacking.

Moreover, following interaction with host cell receptors *Leishmania* spp. become internalized and utilize different mechanisms to infect and establish long term infections within these host cells by effectively suppressing and evading host immune responses. These different mechanisms of evasion may include modification of the complement system and phagocytosis, strategies to survive within phagosomes, interference in antigen presentation, modulation of cytokine and chemokine levels and modification of T cell responses. Additionally, *Leishmania* spp. alter host cell signalling pathways like toll-like receptor pathways, protein kinase C mediated signalling, JAK/STAT pathway activation *via* IFN-γ, MAPK signalling pathway etc. for establishing infection [69]. However, it is also likely that the leishmanial Inl-A-like virulence factors may aid in establishment and/or maintenance of infection by evading or suppressing host immune responses. In this respect one of our collaborators has studied the RNAseq expression profile of intracellular amastigotes isolated from infected murine resident peritoneal macrophages nearly 12 h post-infection. Interestingly in this unpublished work it has been found that the three *L. donovani* Inl-A-like genes are over-expressed when more virulent parasites are used to bring about infection as compared to infection by less virulent parasites. Moreover, the level of E9BMT7 gene shows significant fold change (> 1.5) in these two conditions and therefore it is likely that E9BMT7 or other *L. donovani* Inl-A-like proteins are associated with higher infectivity of *L. donovani.* However, direct experimental evidence elucidating the exact function of these proteins in the amastigote stage is lacking.

Our findings based on sequence and structure analyses raise the possibility that *L. donovani* Inl-A-like proteins may facilitate host cell infection by interaction with host cell receptors (for example E-cadherin). This possibility receives further support from experimental finding such as the leishmanial Inl-A-like proteins being secreted or exported in vesicles by promastigotes present in infected sand fly inocula. Additionally, these proteins could also aid in suppressing/evading host immune responses when expressed in amastigote stage. However, we believe further direct experimental characterization of the *L. donovani* Inl-A-like proteins and identification of their host-protein interaction partners (e.g. E-cadherin) will create new avenues for study. Moreover, in this way if one or more of the *L. donovani* Inl-A-like proteins are proven to be indeed important for the virulence of *Leishmania* spp., we may be able to develop other intervention strategies in leishmaniasis.

## Conclusion

We used extensive sequence-profile and profile-profile comparison-based methods to predict the existence of *L. donovani* Inl-A-like proteins, which are possibly remote orthologs of *L. monocytogenes* Inl-A. We further explored the possibility whether *L. donovani* can adopt similar subversion mechanism in host cells utlizing the Inl-A-like protein-based interaction with human E-cadherin [hEC1]. We addressed this issue based on homology modeling of *L. donovani* Inl-A-like proteins and their subsequent docking studies with the host interaction partner (human E-cadherin [hEC1]) of their bacterial ortholog (Inl-A). Based on these analyses, we suggest the existence of a new group of virulence factor in *L. donovani* and other *Leishmania* spp. capable of employing a yet to be known mode of host invasion mechanism.

## Additional files

Additional file 1: Figure S1. Plot of docking scores against ligand root mean square deviation (l-RMSD) between the crystal complex (PDB ID: 1O6S) and docked poses obtained after re-docking Inl-A with hEC1. (TIF 433 kb)
Additional file 2: Table S1. List of *L. donovani* proteins showing similarity to bacterial virulence factors enlisted in VFDB database [12]. (XLS 33 kb)
Additional file 3: Table S2. Probable Inl-A-like LRR regions within the *L. donovani* Inl-A-like proteins. (XLS 25 kb)
Additional file 4: Figure S2. Ranks of Inl-A-like proteins in Kinetoplastida in the reverse search analysis (identified with the help of a modified forward search [comprising of a BLASTp [15] search against NR database] and a reverse search analysis [comprising of an HHblits [30, 34] search against PDB and UniProt HMM databases]) (a) Orthologs of *L. donovani* Inl-A-like proteins in *Leishmania* spp. (b). (TIF 3054 kb)
Additional file 5: Table S3. Docking results for *L. donovani* Inl-A-like proteins with human E-cadherin (hEC1). (XLS 24 kb)
Additional file 6: Table S4. Probable hydrogen bond forming residues in E9B7L9-hEC1, E9BMT7-hEC1 and E9BUL5-hEC1 complexes. **Table S5.** Probable hydrophobic interaction forming residues in E9B7L9-hEC1, E9BMT7-hEC1 and E9BUL5-hEC1 complexes. (XLS 34 kb)

## Abbreviations

ACP: Atomic contact potential
AIR: Ambiguous interaction restraints
BLAST: Basic local alignment search tool
BSA: Buried surface area
CAPRI: Critical assessment of predicted interactions
c-Evalue: Conditional E-value
CR1: First complement receptor
CR3: Third complement receptor
DARS: Decoys as the reference state
DC-SIGN: Dendritic cell-specific intercellular adhesion molecule-3-grabbing non-integrin
E-value: Expect-value
FcγRs: Fc gamma receptors
FFT: Fast-fourier transform
Fnat: Fraction of native contacts
FnRs: Fibronectin receptors
HADDOCK: High ambiguity driven protein-protein docking
hEC1: Human E-cadherin
HHblits: HMM-HMM–based lightning–fast iterative sequence search
HMM: Hidden Markov Model
i-Evalue: Independent E-value
Inl-A: Internalin-A
l-RMSD: Ligand root mean square deviation
LRR: Leucine rich repeat
MAC: Maximum accuracy
MR: Mannose receptor
NR: Non redundant
PDB: Protein data bank
RMSD: Root mean square deviation
SCOP: Structural classification of proteins
VFDB: Virulence factor database
