# Supplementary figures and images for: Identification of internalin-A-like virulent proteins in Leishmania donovani
Source: Parasit Vectors. 2016 Oct 21;9:557. doi: 10.1186/s13071-016-1842-5 (PMC5073978; doi:10.1186/s13071-016-1842-5)

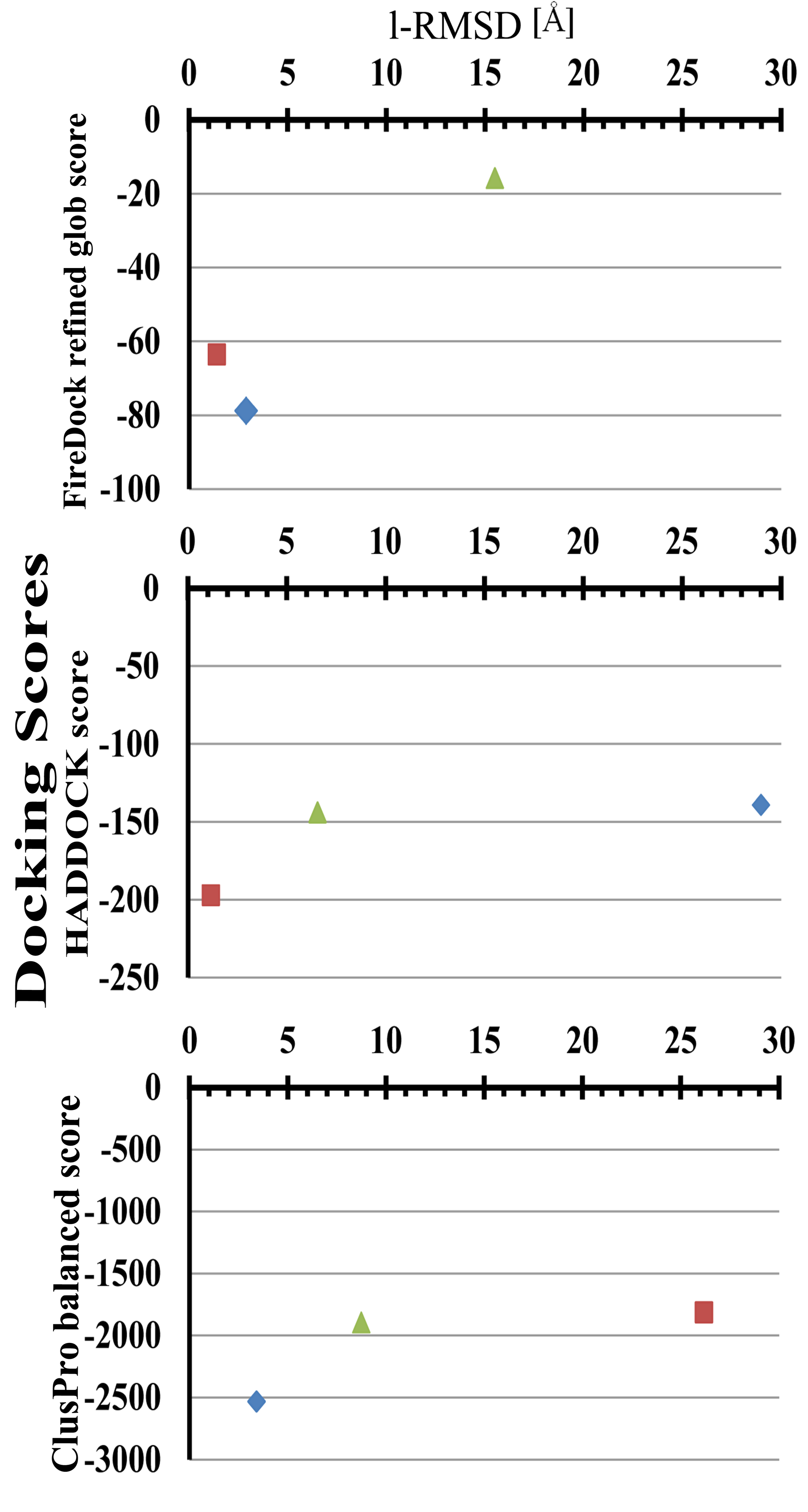

Supplement: Additional file 1: Figure S1. — Plot of docking scores against ligand root mean square deviation (l-RMSD) between the crystal complex (PDB ID: 1O6S) and docked poses obtained after re-docking Inl-A with hEC1. (TIF 433 kb) [file 13071_2016_1842_MOESM1_ESM.tif]

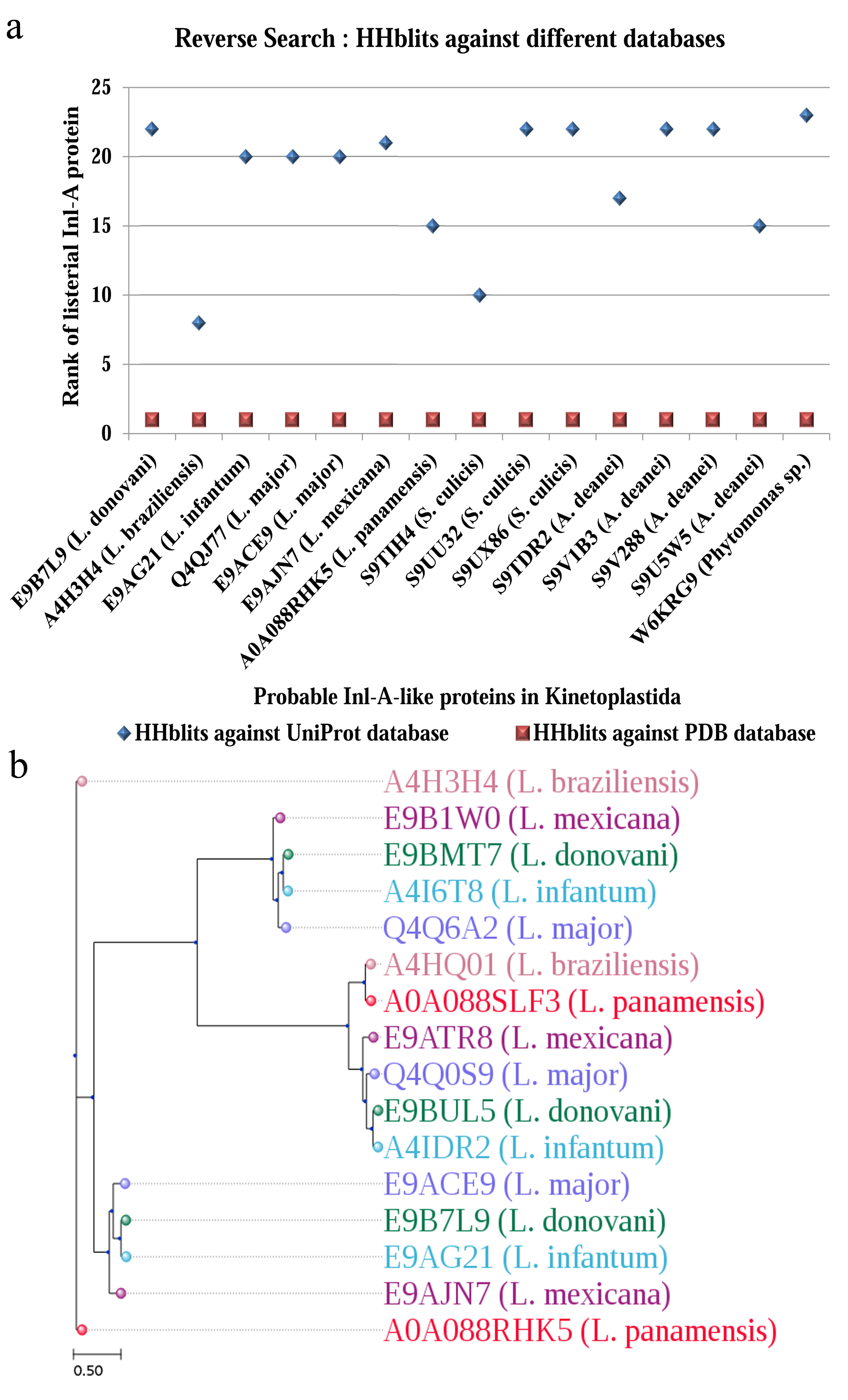

Supplement: Additional file 4: Figure S2. — Ranks of Inl-A-like proteins in Kinetoplastida in the reverse search analysis (identified with the help of a modified forward search [comprising of a BLASTp [15] search against NR database] and a reverse search analysis [comprising of an HHblits [30, 34] search against PDB and UniProt HMM databases]) (a) Orthologs of L. donovani Inl-A-like proteins in Leishmania spp. (b). (TIF 3054 kb) [file 13071_2016_1842_MOESM4_ESM.tif]
